# Dysregulation of hepatic microRNA expression profiles with C*lonorchis sinensis* infection

**DOI:** 10.1186/s12879-016-2058-1

**Published:** 2016-11-30

**Authors:** Su Han, Qiaoran Tang, Xi Lu, Rui Chen, Yihong Li, Jing Shu, Xiaoli Zhang, Jianping Cao

**Affiliations:** 1 Department of Parasitology, Harbin Medical University, Harbin, 150081 China; 2 National Institute of Parasitic Diseases, Chinese Center for Disease Control and Prevention; Key Laboratory of Parasite and Vector Biology, Ministry of Health, MOH; National Center for International Research on Tropical Diseases; WHO Collaborating Center for Tropical Diseases, Shanghai, People’s Republic of China; 3 Department of Gastroenterology, The First Affiliated Hospital of Harbin Medical University, Harbin, China; 4 Department of Orthopedic Surgery, The fourth Affiliated Hospital of Harbin Medical University, Harbin, China

**Keywords:** *Clonorchis sinensis*, Hepatic, MicroRNA, Infection

## Abstract

**Background:**

Clonorchiasis remains an important zoonotic parasitic disease worldwide. The molecular mechanisms of host-parasite interaction are not fully understood. Non-coding microRNAs (miRNAs) are considered to be key regulators in parasitic diseases. The regulation of miRNAs and host micro-environment may be involved in clonorchiasis, and require further investigation.

**Methods:**

MiRNA microarray technology and bioinformatic analysis were used to investigate the regulatory mechanisms of host miRNA and to compare miRNA expression profiles in the liver tissues of control and *Clonorchis sinensis (C. sinensis)*-infected rats.

**Results:**

A total of eight miRNAs were downregulated and two were upregulated, which showed differentially altered expression profiles in the liver tissue of *C. sinensis*-infected rats. Further analysis of the differentially expressed miRNAs revealed that many important signal pathways were triggered after infection with *C. sinensis*, which were related to clonorchiasis pathogenesis, such as cell apoptosis and inflammation, as well as genes involved in signal transduction mechanisms, such as pathways in cancer and the Wnt and Mitogen-activated protein kinases (MAPK) signaling pathways.

**Conclusions:**

The present study revealed that the miRNA expression profiles of the host were changed by *C. sinensis* infection. This dysregulation in miRNA expression may contribute to the etiology and pathophysiology of clonorchiasis. These results also provide new insights into the regulatory mechanisms of miRNAs in clonorchiasis, which may present potential targets for future *C. sinensis* control strategies.

## Background

*Clonorchis sinensis* (*C. sinensis*) is an important food-borne zoonotic parasite that causes an estimated 35 million human infections worldwide primarily throughout countries in eastern and southeast Asia, including China, Japan, Korea, and Vietnam [1, 2]. Human infection occurs by ingestion of insufficiently cooked freshwater fish harboring *C. sinensis* metacercariae. Persistent and chronic infections often lead to induction and progression of hepatobiliary diseases, such as cholangitis, cholelithiasis, cholecystitis, pancreatitis, hepatic fibrosis, cholangiocarcinoma, and liver cancer [3]. However, the precise molecular pathogenic mechanisms in *C. sinensis* infection are not fully understood. Several studies have reported that *C. sinensis* infection induces changes to several biological responses of the host, such as inflammation, cirrhosis, and activation of nuclear factor-κB-mediated inflammation [4–6]. In addition, it is reported that *C. sinensis* excretory-secretory products (ESPs) can induce change to expression profiles of cancer-related microRNAs (miRNAs) in human cholangiocarcinoma cells [7]. However, the modulatory mechanisms of these factors remain to be elucidated.

MiRNAs are a class of endogenous non-coding small RNAs (~22 nucleotides in size) that regulate gene expression at the post-transcriptional level and play important roles in the regulation of diverse physiological and pathological processes, such as cell maintenance, development, proliferation, differentiation, metabolism, and apoptosis [8]. Also, miRNAs are reported to regulate a variety of developmental and physiological processes in various parasites [9], including *Trypanosoma brucei, Toxoplasma gondii*, and *Schistosoma japonicum* [10–12]. Some recent studies have investigated differences in miRNA expression profiles and related specific biological functions in parasite-infected hosts [13]. For example, let-7i regulates Toll-like receptor 4 expression in cholangiocytes and contributes to epithelial immune responses against *Cryptosporidium parvum* infection [14]. Furthermore, *T. gondii* infection was found to induce changes in mouse brain miRNA expression profiles [10]. Hence, studying changes in host miRNA profiles following parasite infection will help to further elucidate host-parasite interactions. The discovery of the functions of miRNAs is expected to provide new insights into better understanding pathogenic mechanisms and control of these parasites.

Considering complex pathogenic mechanism of clonorchiasis*,* it is important to define in detail the host-parasite interaction. MiRNAs act as important mediators of host-parasite interaction and play important roles in host environment. However, knowledge of miRNAs functions after *C. sinensis* infection remains limited. Therefore, the objective of the present study was to analyze miRNA expression profiles in the liver following *C. sinensis* infection using microarray and bioinformatic analyses. The results of this study provide novel comparative information to potentially define the functional significance of host miRNAs, further elucidate the etiology and pathophysiology of clonorchiasis, and provide a better understanding of potential targets for future *C. sinensis* control strategies.

## Methods

### Parasites and hosts

*C. sinensis* metacercariae were harvested from the cyprinid *Pseudorasbora parva* captured in the Songhuajiang River, an endemic area of *C. sinensis* infection in China. Metacercariae were collected as described previously.

Wistar rats (5–6 weeks old, male) were purchased from the Harbin Medical University Laboratory Animal Center (Harbin, China) and housed in an air-conditioned room at 24 °C under a 12-h dark/light cycle with free access to standard laboratory food and water. The rats were individually infected orally with 50 metacercariae. Parasitic infection was assessed by detection of parasite eggs in stool samples and pathological findings. Mock-infected control rats were similarly administered with 50 μl of sterile normal solution. All animal care and experimental procedures were conducted in accordance with the guidelines for animal use in toxicology.

### Total RNA isolation and microarray analysis

The animals were sacrificed at 3 weeks post-infection and the livers were harvested and preserved in TRIzol reagent (Shanghai Invitrogen Biotechnology Co., Ltd., Shanghai, China) at −80 °C, according to the manufacturer’s protocol, until RNA extraction. Total RNA was extracted from the liver tissues of Wistar rats using the mirVana isolation kit (Applied Biosystem p/n AM1556), according to the manufacturer’s protocol. Total RNA was quantified using the NanoDrop ND-2100 spectrophotometer (Thermo Fisher Scientific, Inc., Waltham, MA, USA) and subsequently RNA integrity was assessed using the Agilent 2100 bioanalyzer system (Agilent Technologies, Santa Clara, CA, USA).

Before small RNA isolation, all RNA templates with good integrity of the same group were pooled. The sample labeling, microarray hybridization, and washing were performed in accordance with the manufacturer’s standard protocols. Briefly, total RNA were tailed with Poly A and labeled with biotin. Then, the labeled RNAs were hybridized onto the microarray. After washing and staining the slides, the microarrays were scanned using the Affymetrix Scanner 3000 (Affymetrix). All microarray analyses were performed by Shanghai Oebiotech Co. Ltd., Shanghai, China.

Affymetrix GeneChip Command Console software (version 4.0, Affymetrix) was used to analyze array images to obtain raw data, which was normalized using the robust multiarray average method. Next, Genespring software (version 12.5; Agilent Technologies) was used for the following data analysis. Differentially expressed miRNAs were then identified through fold change as well as the probability (*p*) value calculated using the *t*-test. The threshold set for up- and down-regulated genes was a fold change of ≥ 1.5 and a *p* value of ≤ 0.05. All dysregulated miRNAs were subjected to hierarchical clustering analysis and the results are presented in a heat map.

### MiRNA targeted gene prediction and bioinformatics analysis

Target genes of differentially expressed miRNAs were the intersection predicted with three databases: TargetScan (http://www.targetscan.org/), PITA (http://genie.weizmann.ac.il/pubs/mir07/mir07_data.html), and microRNA.org (http://www.microrna.org/microrna/home.do). Gene ontology (GO) and Kyoto Encyclopedia of Genes and Genomes (KEGG) pathway analyses were applied to determine the roles of the target genes. Hierarchical clustering was performed to distinguish miRNA expression patterns among samples.

### Validation of microarray data by qPCR analysis

Differentially expressed miRNAs were validated using quantitative stem-loop reverse transcription RT-PCR (qPCR) with SYBR green and specifically designed primers. U6 RNA was selected as a housekeeping miRNA for normalization of miRNA expression. The RNA templates for qPCR analysis were obtained from the same samples used for microarray hybridizations. Total RNA from tissues was quantified using a Nanodrop-1000 spectrophotometer and reverse-transcribed to cDNA using RT primers and the SuperScript™ III Reverse Transcriptase kit (Invitrogen Corporation, Carlsbad, CA, USA). Each 20-μL qPCR reaction contained 10 μL of SYBR@ Premix Ex Taq™II polymerase (TaKaRa, Bio, Inc., Dalian, China), 0.8 μL of a forward/reverse primer mixture, 1 μL of cDNA template, and 8.2 μL of Easy Dilution buffer. The cycling protocol was as follows: 95 °C for 30 s, followed by 45 cycles of 95 °C for 20 s, 60 °C for 20 s, and 72 °C for 20 s (single), and then by 1 cycle of 95 °C for 0 s, 65 °C for 5 s, and 45 °C for 5 s. Quantification of each miRNA relative to U6 was calculated using the 2^–ΔΔCt^ method. All assays were performed in triplicate.

### Statistical analysis

Data are expressed as the mean ± standard deviation. Differences in variables between groups were determined using the *t*-test. A *p* value of < 0.05 was considered statistically significant and all statistical analyses were performed using SPSS 11.0 software (IBM-SPSS, Inc., Chicago, IL, USA).

## Results

### Differentially expressed miRNAs

**Fig. 1:**
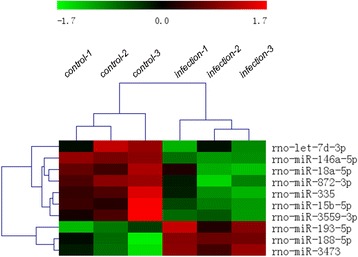
Expression profiling of miRNAs in liver tissue of rats following *Clonorchis sinensis* infection

A total of 728 mature miRNAs sequences of rat liver tissues (Sanger miRbase v17.0) were subjected to miRNA microarray analysis. miRNAs differentially expressed between infected and uninfected animals by an upregulation or downregulation with a signal intensity of >500 are shown in Fig. 1. Log 2 values and fold changes of 10 differentially expressed miRNAs in the livers of Wistar rats infected with *C. sinensis* (two upregulated miRNAs and eight downregulated miRNAs) are presented in Table 1. Among these miRNAs, rno-miR-335 was downregulated the greatest by almost 6-fold.

**Table 1:** The differential expressed miRNAs in liver tissues of Wistar rats infected with *Clonorchis sinensis*

miRNAs	Log2(infected/control)	Fold changes	*p*-value
rno-let-7d-3p	−1.30388	2.468912	0.049085
rno-miR-335	−2.58372	5.994844	0.027821
rno-miR-15b-5p	−0.86776	1.82483	0.03112
rno-miR-18a-5p	−1.97174	3.922417	0.045029
rno-miR-146a-5p	−0.73063	1.659359	5.56E-05
rno-miR-193-5p	0.669321	1.590324	0.015148
rno-miR-872-3p	−1.59381	3.018462	0.014423
rno-miR-188-5p	1.557316	2.943058	0.023138
rno-miR-3559-3p	−1.75129	3.36659	0.031924
rno-miR-3473	0.995508	1.993783	0.018921

### Biological functions of the differentially expressed miRNAs

The main functions of the differentially expressed miRNAs in response to *C. sinensis* infection are shown in Table 2. Most of the differentially regulated miRNAs were annotated as involved in cellular processes, with a direct function of cellular response to stimulus, differentiation, and apoptosis. MiR-335 is reportedly involved in the inhibition of proliferation, migration, and invasion of hepatic stellate cells (HSCs), while miR-146a-5p suppresses activation and proliferation of HSCs in nonalcoholic fibrosing steatohepatitis, and miR-18a-5p increases differentiation of vascular smooth muscle cells.

**Table 2:** Main functions of some differentialy expressed miRNAs in liver tissues of Wistar rats infected with *Clonorchis sinensis*

miRNAs	Fuctions	PMID
rno-miR-335	inhibit the proliferation and migration invasion of HSC cells, target in apoptosis	21586285
rno-miR-15b-5p	target in apoptosis	26119771
rno-miR-18a-5p	increases vascular smooth muscle cell differentiation	25089138
rno-miR-146a-5p	suppresses activation and proliferation of hepatic stellate cells	26537990
rno-miR-188-5p	suppresses tumor cell proliferation and metastasis	25998163

### Prediction of miRNA target genes

We next predicted the functions and the target genes of the differentially expressed hepatic miRNAs following *C. sinensis* infection using KEGG pathway analysis and functional enrichment analysis within the biological process GO categories. These analyses identified only two potential target genes (miR-335 and miR-3559-3p) using the three online software programs (TargetScan, PITA, and microRNA.org) databases. The number of target genes predicted for the differentially expressed miRNAs varied from 8 to 556.

### GO analyses of differentially expressed miRNAs

**Fig. 2:**
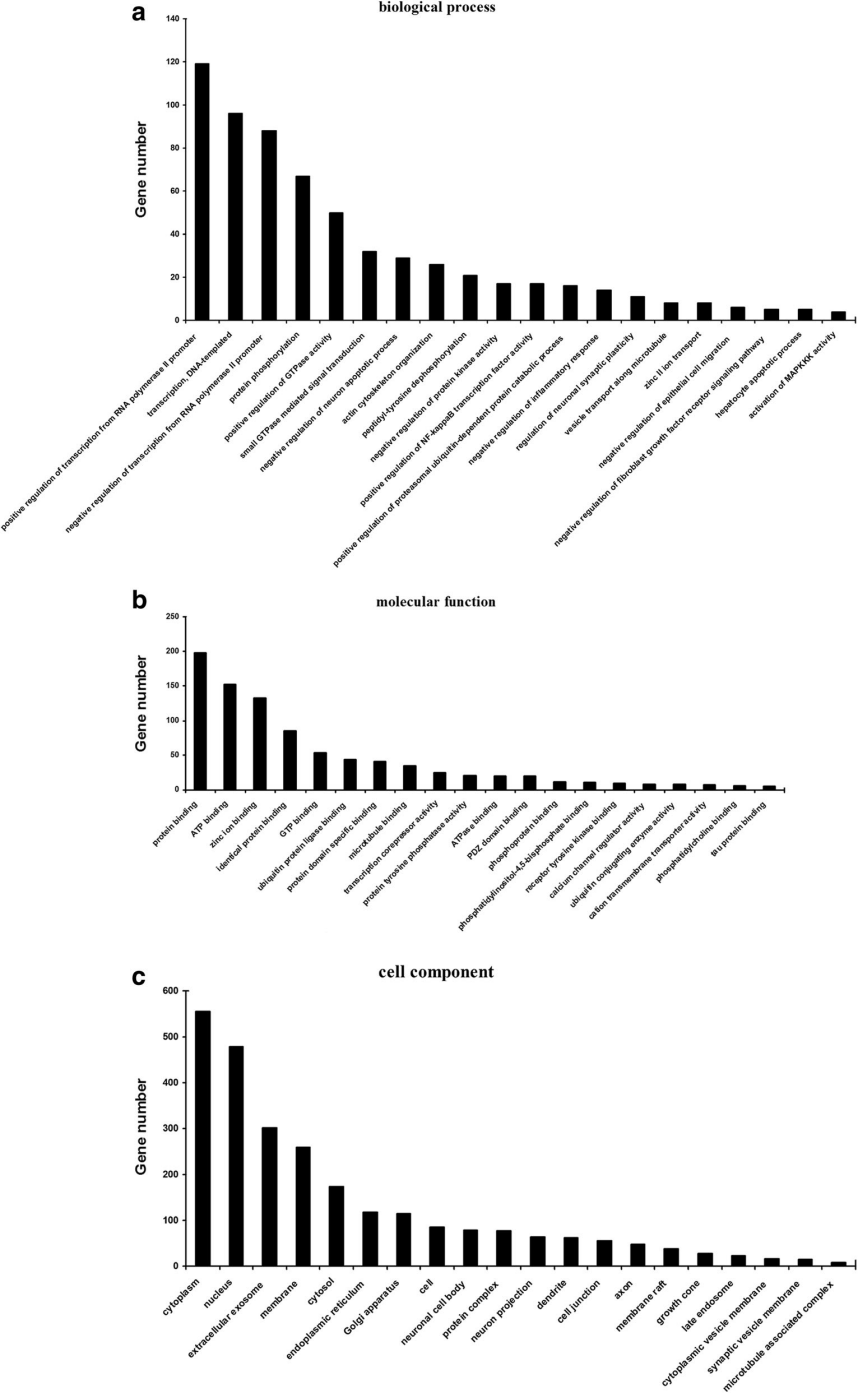
GO analysis of validated targets of differentially expressed miRNAs. According to *P* value, top 30 GO terms of biological process (**a**), molecular function (**b**) and cellular component (**c**) were shown respectively

The predicted target genes were classified according to GO functional annotations. As shown in Fig. 2, there were 20 enriched GO annotations among the predicted target genes of differentially expressed miRNAs. The GO analyses of these predicted target genes revealed that some had potentially important biological functions in the host regarding defense against *C. sinensis* infection. The GO-specific functions were mainly involved in biological processes (e.g., negative regulation of fibroblast growth factor receptor signaling pathway, zinc II ion transport, and protein phosphorylation), cell components (e.g., endoplasmic reticulum, extracellular exosome, and Golgi apparatus), and molecular functions (e.g., protein binding, calcium channel regulator activity, and transcription co-repressor activity).

### Target genes of differentially expressed miRNAs predicted by KEGG pathway analysis

**Fig. 3:**
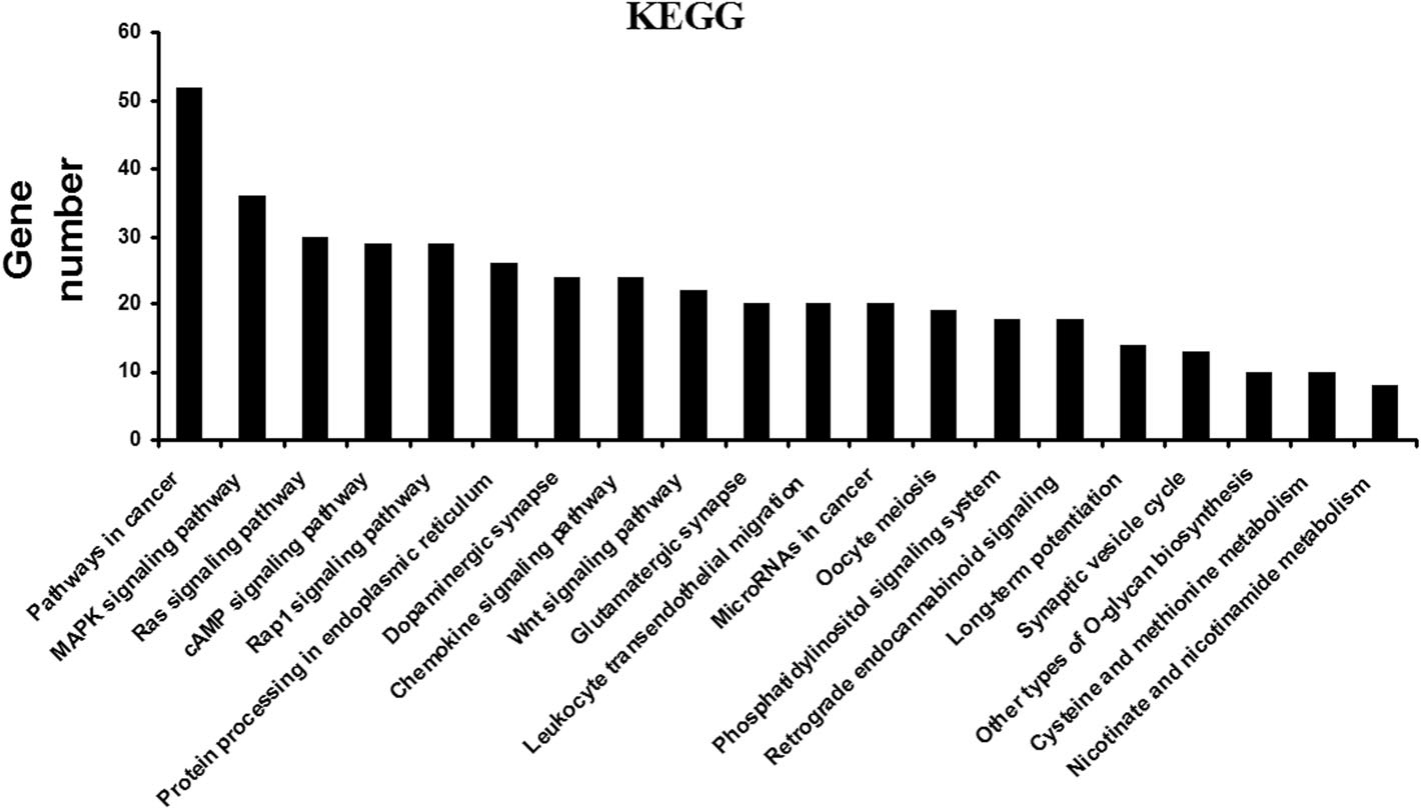
KEGG enrichment analysis of validated targets of differentially expressed miRNAs. Significantly enriched pathways

The KEGG pathway analyses indicated that 10 pathways were subject to regulation by the miRNAs with altered levels, which included pathways involved in cancer progression, phosphatidylinositol signaling, Wnt signaling, and MAPK signaling (Fig. 3). Among these, 52 target genes associated with the altered miRNA levels were involved in cancer signaling and a total of 36 target genes were assigned to the MAPK signaling pathway, which is involved in a wide range of cellular responses, including gene expression, differentiation, proliferation, and apoptosis, as well as growth of malignant tumors. Furthermore, other miRNAs were associated with important pathways in immune regulation, especially chemokine signaling.

### Validation of miRNA microarray data by qPCR analysis

**Fig. 4:**
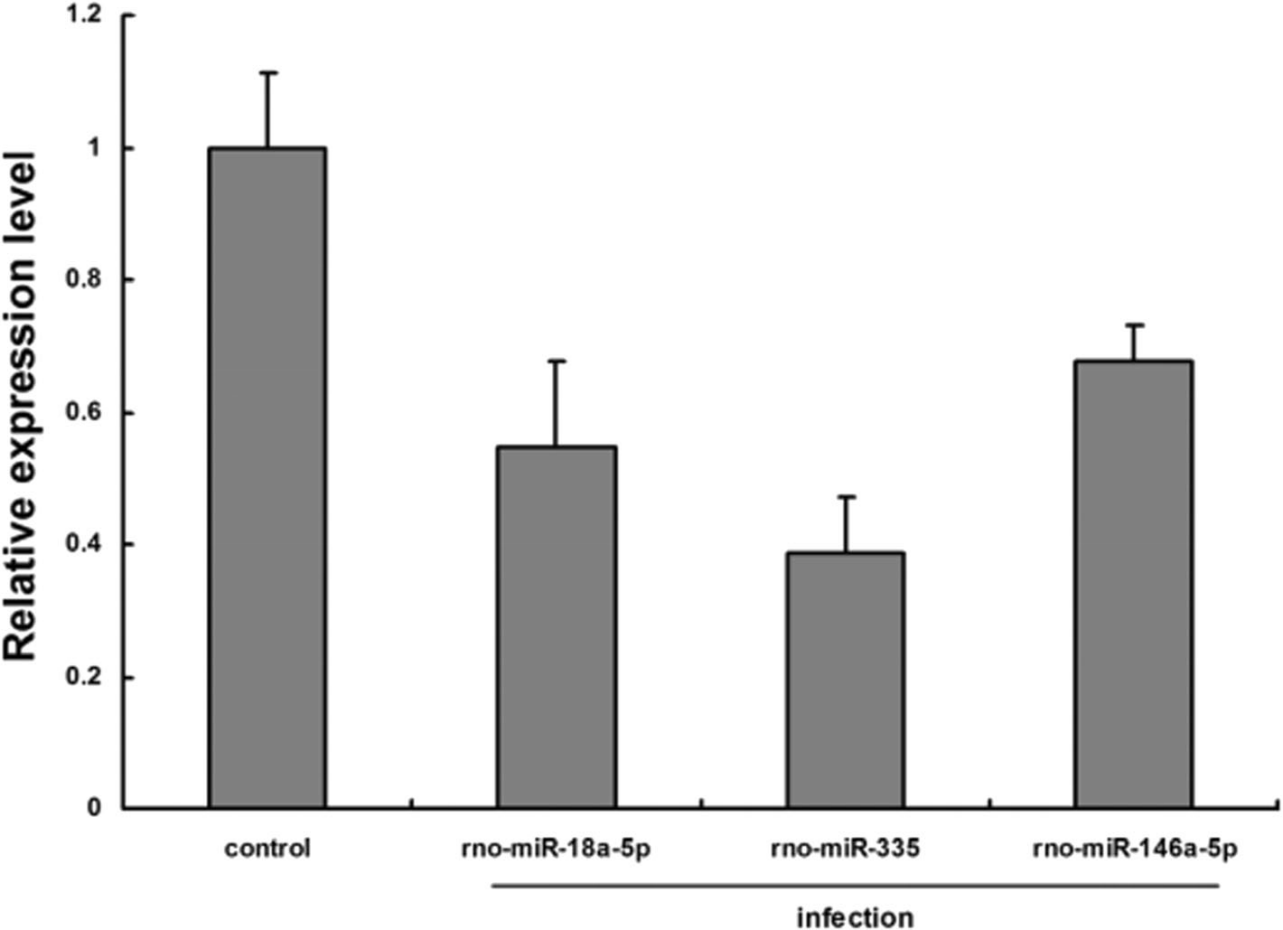
qPCR confirmation of miRNA microarray date subset. Expression rates between various samples are showed by fold change. The data presents the mean and standard error of the mean derived from triplicate experiments

Three selected miRNAs and the housekeeping miRNA miR-U6 were assayed by qPCR to confirm the results of the expression profiles identified by microarray analysis. The expression patterns validated by qPCR agreed with those of the microarray data, which confirmed that miR-335, miR-18a-5p, and miR-146a-5p were expressed in the liver (Fig. 4).

## Discussion

MiRNAs act as key regulators of gene expression at the post-transcriptional level and regulate a variety of biological processes, not only in the normal functioning of eukaryotic cells, but also dysregulation in the pathogenesis, progression, and prognosis of various diseases [15, 16]. miRNAs are essential for the complex life cycles of parasites and play important roles in the abilities of parasites to cause infection [17]. Parasite-host interactions are complicated processes which including many factors. MiRNAs are considered an important player may be involved in the regulation of the molecular mechanism and host micro-environment [9]. The miRNA expression profiles of *C. sinensis* have been investigated and analyzed [18]. However, relatively few studies have investigated differences in miRNA expression patterns and associations with specific biological functions in hosts infected with *C. sinensis*. Therefore, to analyze the interplay between parasites and hosts at the miRNA level, we investigated changes in host miRNA profiles in the livers of Wistar rats 3 weeks post *C. sinensis* infection using microarray technology. The results of these analyses showed that most of the identified miRNAs were similar between infected liver samples and uninfected controls, indicating fundamental regulatory functions of most of these miRNAs. In addition, there were some differentially expressed miRNAs also found in the liver (two miRNAs were upregulated and eight were downregulated), which implied that *C. sinensis* infection altered host gene expression and subsequent changes in miRNA profiles targeting host trans-regulation factors. These specific miRNAs might be involved in pathophysiological processes underlying *C. sinensis* infection*.* These results will help to better elucidate the interplay between parasites and hosts, and further understanding of the host response.

A study has shown that *C. sinensis* infection can induce liver fibrosis [19]. However, the mechanisms underlying infection-induced liver fibrosis remain poorly understood. Activation and migration of resident HSCs within the hepatic space of Disse play important roles in hepatic fibrosis, and miRNAs have been found to play essential roles in HSC differentiation and proliferation. The results of this study showed that miR-146a-5p, which is reported to suppress activation and proliferation of HSCs through directly targeting of the *Wnt1* and *Wnt5a* genes [20], was differently expressed in the liver. miR-335, which is known to downregulate tenascin-C expression, inhibit HSC migration, and reduce the activities of α-SMA and collagen type I [21], was downregulated in the liver of rats infected with *C. sinensis*. Thus, our results suggest that miR-146a-5p and miR-335 might play important roles in the regulation of hepatic fibrosis at the post-transcriptional level following *C. sinensis* infection.

Furthermore, research had shown that *C. sinensis* infection could result in apoptosis of host hepatic cells. Hence, apoptosis may be an important modulator in host-*C. sinensis* interactions and host self-regulation [22]. Our results indicated that the functions of miR-15b-5p and miR-335, which were upregulated in the rat liver following *C. sinensis* infection, include regulation of apoptosis [23, 24]. However, future experiments are required to further reveal the functions of miR-15b-5p and miR-335 during *C. sinensis* infection.

Other than apoptosis, miRNAs also regulate cellular growth, proliferation, metastasis, and differentiation. As shown in the present study, miR-18a-5p was differentially expressed in the livers of infected rats, which suggests that vascular smooth muscle cell differentiation may be altered following *C. sinensis* infection [25]. miRNA-188-5p was significantly decreased in hepatocellular carcinoma, which suppresses tumor cell proliferation and metastasis by directly targeting FGF5 [26]. Therefore, it is tempting to postulate that the downregulation of miR-188-5p partly contributes to the *C. sinensis*-associated induction of cell proliferation during the development of cholangiocarcinoma. It was reported that *C. sinensis* ESPs could induce change to the expression profiles of cancer-related miRNAs in human cholangiocarcinoma cells [7]. However, the identification of specific mechanisms will have to be further explored. Meanwhile, the biological functions of five differentially expressed miRNAs, including miR-193-5p, miR-872-3p, miR-3559-3p, miR-3473, and let-7d-3p, in *C. sinensis* infection need to be further investigated.

The discovery of miRNA function sheds new light on the mechanisms underlying the pathogenesis of *C. sinensis* infection. Therefore, in the present study, GO and KEGG analyses were used to identify reported and predicted target genes involved in *C. sinensis* infection. The most enriched GOs were involved in zinc II ion transport, protein phosphorylation, and extracellular exosome and calcium channel regulator activities. miRNA analysis also identified some target genes that have been implicated in some important pathways and functional biological processes, such as cancer progression, phosphatidylinositol signaling, Wnt signaling, and MAPK signaling. Some GOs involved in the function of host-derived miRNAs in association with parasite infection need to be further studied.

There were some limitations to this study that should be addressed. First, the sample size was relatively limited, thus future comprehensive studies should be conducted to further validate these findings. Second, the target genes and proteins regulated by these altered miRNAs should be further analyzed to better elucidate potential relationships with clonorchiasis.

## Conclusions

Analysis of miRNA expression profiles in the livers of *C. sinensis*-infected Wistar rats demonstrated that dysregulation of miRNAs may contribute to the etiology and pathophysiology of clonorchiasis. The present study provided novel miRNA-based information to increase our understanding of the pathophysiological processes underlying *C. sinensis* infection of the liver of Wistar rats and identified potential targets for future strategies to control *C. sinensis*. Further work needs to investigate for clarifying the direct association of the miRNAs with specific gene function in *C. sinensis* infection.

## Abbreviations

*C. sinensis*: *Clonorchis sinensis*
ESPs: Excretory-secretory products
GO: Gene ontology
KEGG: Kyoto Encyclopedia of Genes and Genomes
MAPK: Mitogen-activated protein kinases
miRNAs: microRNAs
